# The compartmentalization model of nitrate homeostasis: Role of the salivary glands, skeletal muscle, and liver

**DOI:** 10.17179/excli2026-9229

**Published:** 2026-02-24

**Authors:** Asghar Ghasemi, Sajad Jeddi, Khosrow Kashfi

**Affiliations:** 1 Endocrine Physiology Research Center, Research Institute for Endocrine Sciences, Shahid Beheshti University of Medical Sciences, Tehran, Iran; 2 Department of Molecular, Cellular, and Biomedical Sciences, Sophie Davis School of Biomedical Education, City University of New York School of Medicine, New York, NY 10031, USA; 3 Graduate Program in Biology, City University of New York Graduate Center, New York, NY 10091, USA

**Keywords:** nitrate, nitric oxide, skeletal muscle, saliva, circulation, liver, compartmentalization model of nitrate homeostasis

## Abstract

Nitric oxide (NO) is a small, bioactive molecule with diverse physiological functions. It is generated both enzymatically by NO synthases (NOS) and non-enzymatically through the nitrate-nitrite-NO reduction pathway. Recent studies have renewed interest in nitrate-based regulation of NO, highlighting a compartmentalization model of nitrate homeostasis. In this model, excess NO is rapidly oxidized to nitrite, which is then oxidized to nitrate, a more stable species that limits oxidative damage. Nitrate is differentially distributed across tissues, allowing both storage and rapid mobilization to maintain NO availability. Human and animal studies show that nitrate concentrations in skeletal muscle, plasma, and liver are approximately 100, 35, and 10 nmol/g, respectively, corresponding to a skeletal muscle-to-plasma-to-liver ratio of ~3:1:0.3. The large skeletal muscle reservoir and its higher muscle-to-plasma gradient support the release of nitrate into the circulation when needed. In contrast, the liver-to-plasma ratio < 1 suggests active hepatic uptake of circulating nitrate. Together, these findings support a compartmentalized system in which nitrate storage and flux contribute to whole-body NO homeostasis. Understanding this model may have implications for exercise physiology, metabolic regulation, and liver pathophysiology - all conditions in which NO biology plays a critical role.

See also the graphical abstract(Fig. 1).

## Introduction

There are two major pathways for nitric oxide (NO) production in the human body (Ghasemi, 2022[29]): (1) the canonical *L*-arginine-NO pathway in which different isoforms of NO synthase (NOS), including endothelial (eNOS), neural (nNOS), and inducible (iNOS) isoforms, act to convert *L*-arginine to NO and *L*-citrulline; and (2) the complementary nitrate-nitrite-NO pathway in which nitrate is sequentially reduced to nitrite and then to NO. In addition to endogenous production, the nitrate-nitrite-NO pathway can be enhanced by dietary intake; this is important because it represents a simple and accessible strategy for preventing chronic disease (Lundberg et al., 2018[66]). In particular, vegetables, which play a preventive role in chronic disease (Boeing et al., 2012[15]), are the primary dietary source of nitrate (Ghasemi and Jeddi, 2017[30]).

The two NO production pathways are interconnected and operate simultaneously to meet the body's NO requirements (Piknova et al., 2024[86]). The cyclicity concept of NO production and storage (Piknova et al., 2025[83]), together with the spatial compartmentalization model of nitrate (Park et al., 2021[80]; Piknova et al., 2021[87]) proposed by Piknova et al., suggests that NOS enzymes produce NO, which can subsequently be oxidized to nitrate and transported to other tissues (Piknova et al., 2025[83]). Transported and ingested nitrate can be stored in tissues, where it can be reduced back to NO (Piknova et al., 2025[83]). Thus, by its conversion to nitrate, NO is prevented from causing unwanted and potentially oxidative damage, while the storage of abundant amounts of inert nitrate ensures rapid and uninterrupted access to NO when needed (Piknova et al., 2025[83]).

Following intestinal absorption, nitrate is distributed by blood, which serves as the main transport medium and distribution route for nitrate (Piknova et al., 2024[86]; Piknova et al., 2021[87])), to various tissues, particularly the salivary glands (which concentrate nitrate and participate in the nitrate enterosalivary circulation (Ghasemi, 2022[29])), skeletal muscle (the primary site of nitrate production (Piknova et al., 2015[84]) and the principal site of renewable nitrate storage (Piknova et al., 2025[83])), and the liver (as the principal site of nitrate reduction (Piknova et al., 2025[83]; Piknova et al., 2024[86])). This review provides a closer examination of the compartmentalization model of nitrate homeostasis by summarizing evidence from human and animal studies.

## Nitrate Homeostasis: An Overview

**Figure 2:**
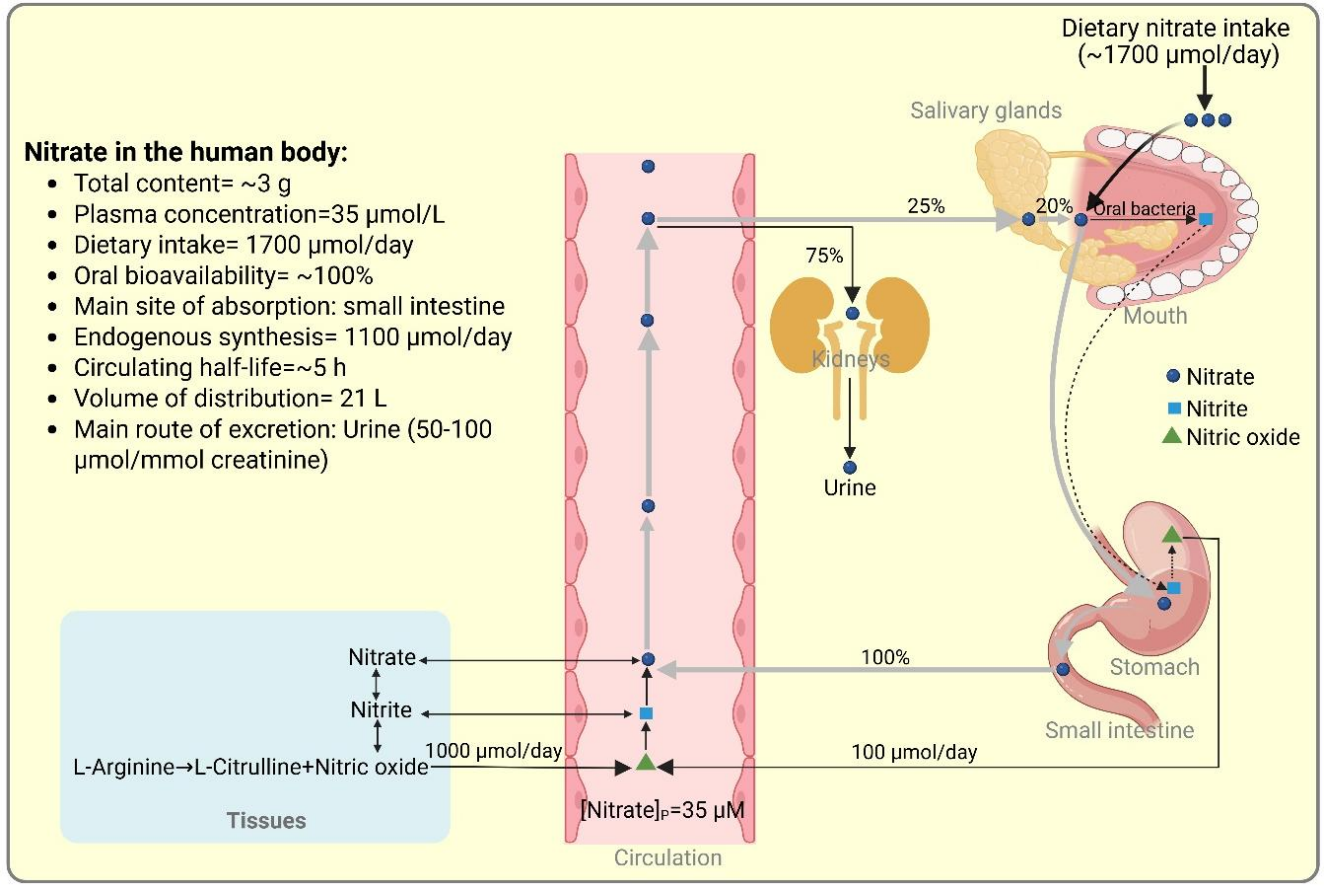
An overview of nitrate homeostasis in the human body. Created in https://BioRender.com

Previous estimates of the total body content of nitrate in a 70-kg human were approximately 400-1,000 mg (Packer and Leach, 1991[76]; Witter et al., 1979[122]); however, following recent findings that skeletal muscle, bone, and skin store substantial amounts of nitrate, this estimation has been revised upwards to approximately 3 g (Piknova et al., 2024[86]). As shown in Figure 2(Fig. 2), the major sources of nitrate in the human body are the oxidation of NOS-derived NO [62 mg/day (~1,000 μmol/day (Ghasemi, 2022[29]))] and dietary sources [108 mg/day (~1,700 μmol/day (Babateen et al., 2018[2]))]. Ingested nitrate is rapidly and efficiently absorbed from the intestine (Cortas and Wakid, 1991[20]; Hezel and Weitzberg, 2015[41]; Kadach et al., 2022[51]; van Velzen et al., 2008[112]; Villar et al., 2021[113]), and its oral bioavailability is approximately 100 % (van Velzen et al., 2008[112]). The mechanism of nitrate absorption from the gastrointestinal tract remains unclear and warrants further investigation.

After absorption, the salivary glands actively take up nitrate from the circulation and secrete it into saliva as part of the enterosalivary circulation. Approximately 25 % of ingested nitrate is taken up by the salivary glands, of which about 20 % is converted to nitrite in the oral cavity and subsequently to NO in the stomach (~5 % of ingested nitrate), providing approximately 100 μmol/day NO to the human body, compared with approximately 1,000 μmol/day produced by NOS enzymes (Ghasemi, 2022[29]; Hezel and Weitzberg, 2015[41]).

Nitrate has a circulating half-life of approximately 5 h (Wagner et al., 1983[114]), a volume of distribution of 21 L (Wagner et al., 1983[114]), and is primarily excreted in the urine (50-90 %, average ~75 %) (Ghasemi, 2022[29]). Following ingestion of potassium nitrate (12.8 mmol, ~1,300 mg), urine nitrate increased from its basal value (1,720 nmol/g) by 3.4-fold (to 5,840 nmol/g) within 1 hour and peaked between 3-9 hours (5-fold, 8,580 nmol/g) and returned to near baseline values (2,600 nmol/g) after 24 hours (Kadach et al., 2022[51]). Basal creatinine-corrected nitrate excretion rate in the urine is 50-100 μmol/mmol creatinine (Tsikas, 2008[108]). Renal clearance of nitrate in healthy humans is approximately 20 mL/min (Wennmalm et al., 1993[120]), but is diet-dependent, ranging from 20 to 125 mL/min (Bahadoran et al., 2021[5]; Wagner et al., 1983[114]; Weinberg et al., 2006[118]; Wennmalm et al., 1993[120]). Very high concentrations of nitrate in urine (up to about 3,000 μM) have been reported (Tsikas et al., 2006[109]). These data indicate that renal nitrate clearance can be lower or higher than GFR (i.e., 125 mL/min in normal conditions), highlighting the kidney's role in regulating nitrate homeostasis.

## Nitrate and Chronic Disease Prevention

Research on the role of nitrate in health and disease suggests that dietary nitrate is a potential health-promoting compound and that nitrate intervention can be considered a nutrition-based strategy for preventing chronic diseases. Inorganic nitrate may play a role in preventing cardiovascular diseases (Kapil et al., 2020[52]; Lundberg et al., 2011[65]; Omar et al., 2016[75]), metabolic disorders, including obesity, metabolic syndrome, and type 2 diabetes (Ghasemi and Jeddi, 2017[30]; Lundberg et al., 2018[66]; Omar et al., 2016[75]), and chronic kidney disease (Jeddi et al., 2025[47]). In addition, nitrate may participate in the prevention of complications of type 2 diabetes, including diabetic nephropathy (Jeddi et al., 2025[47]) and diabetic neuropathy (Bahadoran and Ghasemi, 2025[3]). Results of systematic reviews and meta-analyses partly confirm this conclusion [see Sokal-Dembowska et al. for a recent review (Sokal-Dembowska et al., 2025[98])]. Results of systematic reviews and meta-analyses of randomized controlled trials indicate that inorganic nitrate consumption is associated with improved endothelial function (Bahrami et al., 2021[9]; Celik et al., 2026[19]) and decreased arterial stiffness (Bahrami et al., 2021[9]); inorganic nitrate increased flow-mediated dilation by 1 %, which translated to 13 % risk reduction of adverse CVD events; authors have concluded that nitrate has therapeutic potential for CVD prevention (Celik et al., 2026[19]).

Intervention with a natural source of nitrate has been suggested as a cost-effective dietary approach to enhance NO signaling and prevent chronic diseases (Lundberg et al., 2018[66]; Lundberg et al., 2015[67]; Qin and Wang, 2022[90]). Unlike organic nitrates that produce nitrate tolerance and cause endothelial dysfunction, inorganic nitrates are not associated with nitrate tolerance and improve endothelial function (Qin and Wang, 2022[90]). However, despite the promising effects of inorganic nitrate in preventing chronic diseases, research on inorganic nitrate is still in the preclinical stage (Qin and Wang, 2022[90]). Determining the optimal nitrate dose for intervention, the source of nitrate to be used, and the duration of intervention are among issues that still need further investigation. Dietary data from 52,247 participants of the Danish Diet, Cancer and Health Study over 27 years indicate that intake of plant-sourced nitrate is associated with reduced risk of all-cause mortality (13 %). In contrast, animal-source nitrate (9 %), additive permitted meat-sourced nitrate (19 %), and tap water-sourced nitrate (19 %) were associated with increased risk of all-cause mortality (Bondonno et al., 2024[16]). Furthermore, plant-sourced nitrate has been proposed as a conditionally essential nutrient for cardiovascular health (Pinaffi-Langley et al., 2024[88]). In addition, a recent study in mice indicates that long-term nitrate administration (sodium nitrate, 1 mM in drinking water for 1 year) is ineffective in preventing metabolic consequences of an obesogenic (high-fat, high-sucrose) diet; in addition, it was associated with steatotic liver disease progression, acceleration to hepatocellular carcinoma, increasing cardiac fibrosis, and worsened metabolism (Sowton et al., 2025[99]).

**Figure 3:**
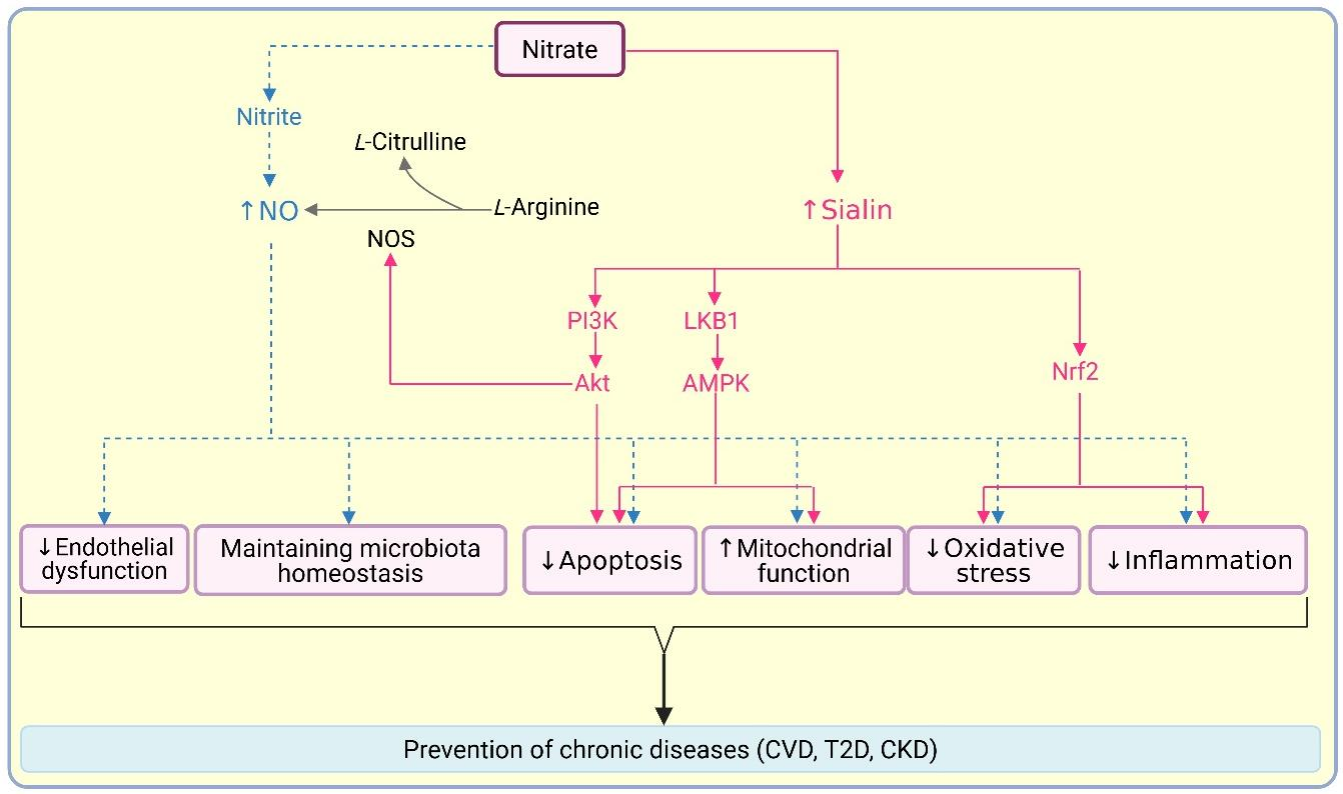
Role of nitrate in preventing chronic diseases. Nitrate contributes to chronic disease prevention by increasing nitric oxide (NO) production via the nitrate-nitrite-NO pathway (dashed blue lines) and by increasing sialin (continuous red lines). Akt, protein kinase B; AMPK, adenosine monophosphate (AMP)-activated kinase; CKD, chronic kidney disease; CVD, cardiovascular disease; LKB1, liver kinase B1; NOS, NO synthase; Nrf2, nuclear factor erythroid 2-related factor 2; PI3K, phosphatidyl inositol-3-kinase; T2D, type 2 diabetes. Created in https://BioRender.com

Positive effects of nitrate therapy in the prevention/treatment of chronic diseases are due to two main mechanisms: (1) NO-dependent effects via the nitrate-nitrite-NO pathway, (2) NO-independent effects by increasing sialin expression and cleavage. The prevailing view is that the positive effects of nitrate are mainly due to the formation of NO, which increases NO bioavailability, improves endothelial function, improves energy metabolism and mitochondrial function, promotes microbiota homeostasis, and exerts anti-hypertensive, anti-oxidant, anti-inflammatory, and anti-apoptotic effects (Bahadoran et al., 2021[4]; Membrino et al., 2025[70]; Qin and Wang, 2022[90]; Sokal-Dembowska et al., 2025[98]) (Figure 3(Fig. 3)). However, recent evidence indicates that nitrate also has NO-independent effects, increasing sialin (Li et al., 2025[59]; Li et al., 2025[60]), which is widely expressed across tissues (Reimer, 2013[92]). The predominant subcellular location of sialin is the lysosomal membrane (Reimer, 2013[92]), where it effluxes sialic acid from lysosomes (Reimer, 2013[92]). Plasma membrane sialin acts as a nitrate transporter (Qin et al., 2012[89]). Nitrate triggers proteolytic cleavage of plasma membrane sialin (~54 kDa), by lysosomal and proteasomal cathepsin B to generate sialin2 (~31 kDa), which acts as a nitrate sensor (Li et al., 2025[61]). Sialin2 is preferentially localized to mitochondria and partially distributed in lysosomes, endoplasmic reticulum, and Golgi apparatus (Li et al., 2025[61]), and explains how nitrate directly and independently of nitrite and NO affects signaling in mammals (Li et al., 2025[60]). It has been proposed that when intracellular nitrate is low, plasma membrane sialin is upregulated which increases transmembrane nitrate import. However, when intracellular nitrate is high, it is cleaved to sialin2, which ceases nitrate uptake, functions as a nitrate sensor, and participates in signal transduction pathways to sustain metabolic homeostasis (Li et al., 2025[61]).

Nitrate activates sialin2-liver kinase B1 (LKB1)-AMP-activated protein kinase (AMPK) signaling pathway in HEK293T cells (human embryonic kidney cell line); AMPK, a master regulator of cellular energy state (Xu et al., 2014[126]), increases mitochondrial biogenesis and suppresses apoptosis (Li et al., 2025[61]). In the liver, nitrate exerts NO-independent anti-inflammatory effects; nitrate increases sialin expression, which increases nuclear factor erythroid 2-related factor 2 (Nrf2). Nrf2, a transcription factor and master regulator of the cellular defense system against oxidative stress (Lee et al., 2005[56]), increases the polarity of liver macrophages toward an anti-inflammatory phenotype, thereby ameliorating liver metabolic dysfunction (Li et al., 2025[59]). Chronic inflammation is associated with metabolic syndrome, obesity, type 2 diabetes, and neurodegenerative diseases (Sokal-Dembowska et al., 2025[98]). Therefore, these findings are relevant regarding the prevention of chronic diseases. In addition, nitrate, via sialin2 and phosphatidyl inositol-3-kinase (PI3K)-protein kinase B (Akt)-NO synthase (NOS) signaling, stimulates NO production in endothelial cells and suppresses apoptosis (Li et al., 2025[60]), indicating a reduction-independent pathway for NO production from nitrate. In addition to nitrate (Feng et al., 2021[26]; Park et al., 2023[79]; Wang et al., 2025[115]; Wang et al., 2024[116]; Xu et al., 2024[127]), sialin gene expression increases by hypoxia (Xu et al., 2024[127]; Yin et al., 2006[129]), inflammation (Akhtar et al., 2024[1]), and type 2 diabetes (Yousefzadeh et al., 2023[130]), and decreases by age (Li et al., 2018[58]; Piknova et al., 2023[85]; Wang et al., 2024[116]), indicating how nitrate can affect whole body homeostasis (see Zhou et al. (Zhou et al., 2024[131]) for a recent review).

In summary, nitrate, particularly from natural sources such as vegetables, can be used as a nutrition-based strategy for health promotion and disease prevention. In addition to increasing NO bioavailability, recent evidence indicates that nitrate has NO-independent beneficial effects achieved via the sialin system. However, further research is needed to elucidate NO-independent effects of nitrate in the human body.

### Epigenetic potential of dietary nitrate for the prevention of chronic diseases

Epigenetics is generally defined as reversible changes in gene expression that are not due to changes in DNA sequence (Lacal and Ventura, 2018[55]; Ling and Rönn, 2019[62]). According to the Minkowskian cone view, assuming conception as the zero point, indirect epigenetics includes all adaptations in parental life that precede conception (across indirect epigenetics) or occur during the gestational period (within indirect epigenetics), and direct epigenetics includes plastic processes that can occur after birth (Lacal and Ventura, 2018[55]). Epigenetic inheritance refers to the transmission of certain epigenetic changes to offspring, and its central concept is that “information about the environment is passed to the next generation” (Lacal and Ventura, 2018[55]).

Modifications to chromatin density (DNA methylation, acetylation, and phosphorylation), histone modification, and non-coding RNAs (ncRNAs) are major mechanisms underlying epigenetic inheritance (Lacal and Ventura, 2018[54]; Suárez et al., 2023[104]). DNA methylation, the primary mechanism of epigenetic inheritance, is the covalent binding of a methyl group to the cytosine residue that is catalyzed by DNA methyltransferases (DNMTs) and alters gene expression; methylation at the gene promoter is associated with gene silencing, and methylation of the transcribed region increases transcriptional activity (Lacal and Ventura, 2018[55]). ncRNAs, that are not translated to proteins, may have < 200 nucleotides (short or small ncRNAs, sncRNAs) or > 200 nucleotides (long ncRNAs, lncRNAs) (Lacal and Ventura, 2018[55]). ncRNAs participate in epigenetic regulation through different mechanisms, including transcriptional regulation, RNA stability, and protein complex recruitment (Lacal and Ventura, 2018[55]).

**Figure 4:**
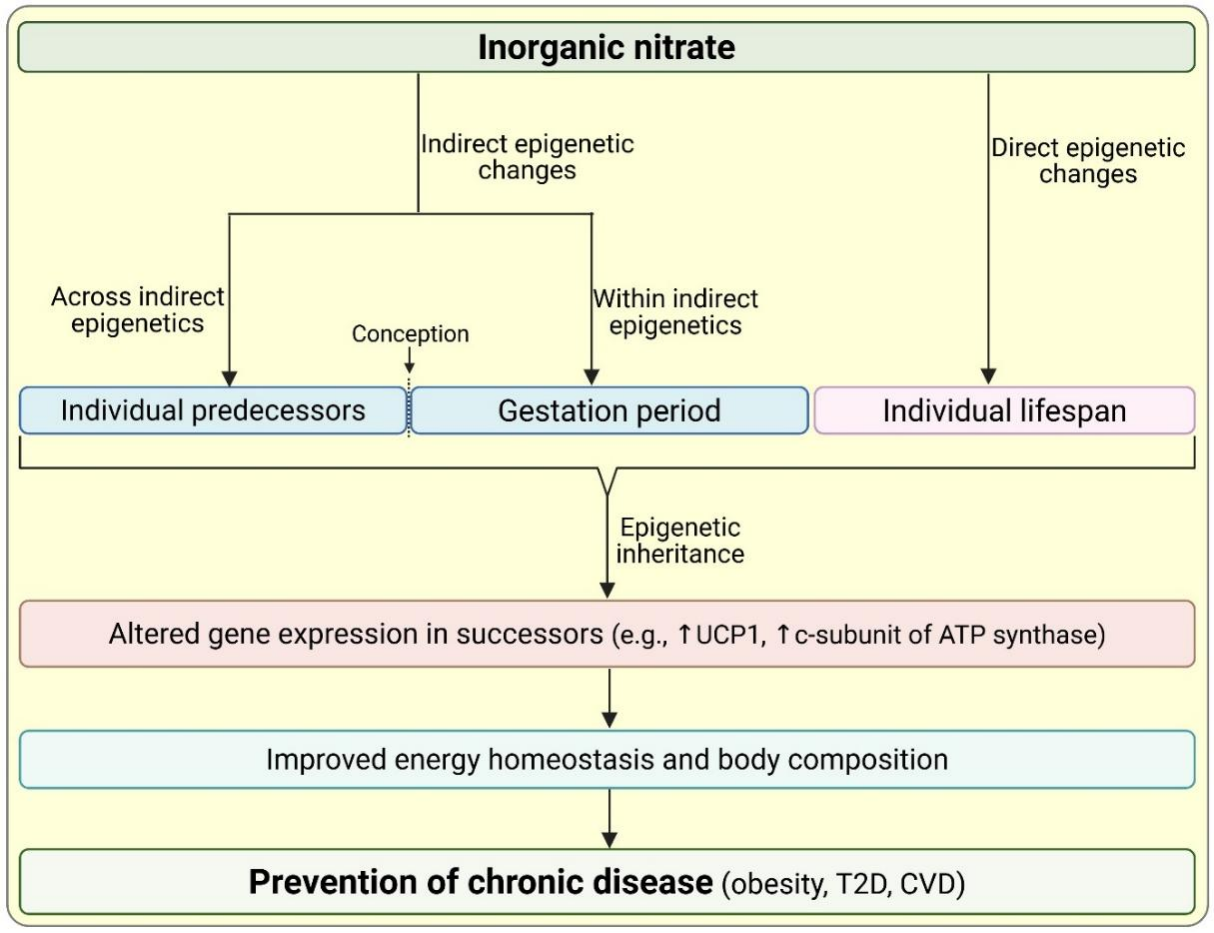
Proposed illustration indicating the contribution of nitrate by epigenetic changes and epigenetic inheritance in the prevention of chronic diseases. See text for details. CVD, cardiovascular disease; T2D, type 2 diabetes; UCP1, uncoupling protein 1. Created in https://BioRender.com

Epigenetic dysregulation contributes to many diseases, including obesity, type 2 diabetes, and cardiovascular diseases (Lacal and Ventura, 2018[55]; Ling and Rönn, 2019[62]; Suárez et al., 2023[104]; Yang et al., 2011[128]). Diet is one of the factors that trigger epigenetic changes (Lacal and Ventura, 2018[55]; Tzika et al., 2018[110]), and a healthy diet helps prevent non-communicable diseases through epigenetic mechanisms (Ling and Rönn, 2019[62]). The contribution of inorganic nitrate to the prevention of chronic diseases through epigenetic inheritance has been addressed in a few animal (Crowe-White et al., 2025[21]; Serrano-Nascimento, 2021[95]) and human (Gonzalez-Nahm et al., 2017[37]; Jönsson et al., 2021[48]) studies, which will be discussed below and summarized in Figure 4(Fig. 4).

It has been reported that high maternal adherence to a Mediterranean Diet is associated with hypomethylation of the maternally expressed gene 3:intergenic differentially methylated region (MEG3-IG MDR) in offspring, which may be protective against type 2 diabetes (Gonzalez-Nahm et al., 2017[37]). Results of a genome-wide epigenetic analysis of cord blood from pregnant women with obesity indicate that adherence to a lifestyle intervention (physical activity with or without the Mediterranean Diet) during pregnancy is associated with greater lean mass in offspring (Jönsson et al., 2021[48]). A recent study by Crowe-White et al. report that nitrate supplementation (40 mg/day translated to 600 mg/day in humans that is provided by the DASH diet) in the HFD-induced obese female rats during the periconceptional (4 weeks before mating) and prenatal (during pregnancy) window has chronic imprinting effects on offspring (Crowe-White et al., 2025[21]); Compared to offspring from mothers on HFD, those from mothers on HFD + nitrate had lower fat mass, lower serum glucose, and lower serum triglycerides at post-natal day 65. In addition, maternal HFD decreased the protein expression of uncoupling protein 1 (UCP1) and c-subunit of mitochondrial ATP synthase in the brown adipose tissue of offspring, which decreases energy expenditure. Treatment of mothers with nitrate increased the expression of both proteins, indicating the epigenetic effect of nitrate on energy expenditure (Crowe-White et al., 2025[21]). It should be noted that epigenetic modifications are tissue- and even cell-specific; thus, it is important to study tissues relevant to a particular intervention (Ling and Rönn, 2019[62]). Nitrate is a competitive inhibitor of iodide uptake by thyroid; it has been shown that nitrate administration (20 and 50 mg/L in drinking water) to pregnant mice increases histone methylation and decreases histone acetylation, resulting in decreased expression of thyroid transcription factors and thyroid differentiation markers and disrupts thyroid development, as measured at gestational day 16.5 (Serrano-Nascimento, 2021[95]).

In summary, epigenetics links environmental factors (such as dietary habits) to altered gene activity and disease phenotypes (such as obesity) in the next generations (Ling and Rönn, 2019[62]). Epigenetic modulation has the potential to play a significant role in preventing non-communicable diseases. Further studies are needed to demonstrate the potential of dietary nitrate for preventing non-communicable diseases through epigenetic mechanisms.

## NO Production in the Salivary Glands, Skeletal Muscle, and Liver

### Salivary glands

All three NOS isoforms are expressed in salivary glands. nNOS has been found in human salivary duct epithelium (Soinila et al., 2006[97]), rat parotid gland acinar cells (Mitsui and Furuyama, 2000[71]), rat submandibular gland acinar and duct cells (Xu et al., 1997[125]), and to a lesser extent, duct cells in rabbits (Sugiya et al., 2001[105]). eNOS has been found in the human salivary duct epithelium (Bentz et al., 1998[13]; Soinila et al., 2006[97]). iNOS has been reported in the human salivary duct epithelium (Brennan et al., 2000[17]) and in rat submandibular gland acinar and duct cells (Xu et al., 1997[125] ). Collectively, these findings suggest that nNOS is the main NO-producing NOS isoform in the salivary glands and also highlight species-dependent differences in NOS expression (Hezel and Weitzberg, 2015[41]; Looms et al., 2002[64]; Soinila et al., 2006[97]). However, data on NOS expression in salivary glands are not entirely consistent. For example, it has been reported that none of the NOS isoforms are expressed in human salivary gland acinar cells (Bentz et al., 1998[13]; Soinila et al., 2006[97]), and that eNOS and iNOS are not expressed in rat submandibular gland acinar and duct cells (Xu et al., 1997[125]).

Despite nitrate uptake at physiological extracellular nitrate concentrations (50-1,000 μM), human submandibular gland cells produce NO and cyclic guanosine monophosphate (cGMP) only at high, non-physiological nitrate concentrations (≥ 15 mM). This contrasts with the liver, where a much lower extracellular nitrate concentration (500 μM) increases intracellular NO production, indicating that, under physiological conditions, salivary gland cells primarily accumulate nitrate for transport and concentration in saliva rather than for NO generation (Qin et al., 2012[89]). Another factor supporting the limited contribution of nitrate-derived NO in the salivary glands is the low nitrate reductase activity in these glands. In eukaryotic cells, the enzyme xanthine oxidoreductase (XOR) catalyzes the reduction of nitrate to nitrite, which can subsequently be reduced to NO under physiological conditions (Jansson et al., 2008[46]). XOR gene expression in the salivary glands (3.8 TPM, transcript per million) is intermediate between that of the liver (26.6 TPM) and skeletal muscle (0.08 TPM), as indicated by data from the Genotype-Tissue Expression (GTEx) portal (https://gtexportal.org).

In mammals, XOR exists in two interconvertible forms derived from the same gene product: xanthine dehydrogenase (XDH) and xanthine oxidase (XO) (Hille and Nishino, 1995[42]). Under metabolic or oxidative stress, XDH can be post-translationally converted to XO (Hellsten et al., 1996[40]; Williams et al., 2023[121]). Both forms catalyze the oxidation of hypoxanthine to xanthine and xanthine to uric acid, but XDH and XO use NAD^+^ and O_2_ as electron acceptors, respectively (Hille and Nishino, 1995[42]). Total XOR activity in mouse liver (3.3 nmol/min/mg protein) is approximately twofold higher than in salivary glands (1.6 nmol/min/mg protein); however, the proportion of the enzyme present in the XO form is substantially higher in the salivary glands (73 %) than in the liver (14 %) (Kusano et al., 2023[54]). This high XO activity in salivary glands contrasts with most other tissues, where XOR is predominantly expressed as XDH (Kusano et al., 2023[54]), the predominant form in vivo (Godber et al., 2001[35]). Importantly, XDH is about 50 times more efficient than XO in reducing nitrite to NO (Godber et al., 2000[36]). Collectively, these data indicate minimal, if any, conversion of nitrate to NO in the salivary glands.

### Skeletal muscle

NO production in skeletal muscle is mainly NOS-dependent. In support of this, decreased NO release has been reported following NOS inhibition in resting diaphragm fibers (67 %, from 2.8 to 0.9 pmol/min/mg muscle) (Kobzik et al., 1994[53]; Reid et al., 1998[91]) and extensor digitorum longus (EDL) muscle (68 %, from 1 to 0.3 pmol/min/mg muscle) (Balon and Nadler, 1994[11]) in male Sprague-Dawley rats, as well as in tibialis anterior (TA) skeletal muscle (57-77 %) in rabbits (Sutherland et al., 2001[106]).

Human skeletal muscle fibers express all three isoforms of NOS, with nNOS being the most abundant isoform (Bahadoran et al., 2024[7]; Frandsen et al., 1996[28]; Stamler and Meissner, 2001[101]), and its mRNA expression is even higher in human skeletal muscle than in the brain (Nakane et al., 1993[72]). Under physiologic conditions, nNOS appears to be the principal NOS isoform responsible for NO production in skeletal muscle (Baldelli et al., 2014[10]), with little or no contribution from eNOS (Gilliard et al., 2018[34]; Hirschfield et al., 2000[43]) or iNOS (Sutherland et al., 2001[106]). In support of this notion, the rate of NO release from diaphragm (14.9 ± 9.1 vs. 18.6 ± 4.8 pmol/min/mg muscle) or soleus muscle (12.8 ± 6.8 vs. 15.6 ± 2.2 pmol/min/mg muscle) did not differ between wild-type and eNOS-deficient mice (Hirschfield et al., 2000[43]), indicating that eNOS does not contribute to resting NO release from skeletal muscle (Hirschfield et al., 2000[43]) or may have only a minor role (Gilliard et al., 2018[34]). Furthermore, Ca^2+^ removal completely blocked NO production in TA muscle in rabbits, indicating that iNOS does not contribute to NO production in skeletal muscle under normal conditions (Sutherland et al., 2001[106]). Consistent with these findings, GTEx portal data indicate that mRNA expression of nNOS in skeletal muscle is about 5-fold and 35-fold higher than that of eNOS and iNOS, respectively.

In skeletal muscle, nitrate can also be reduced to NO. The presence of both XOR and aldehyde oxidase (AO), which are molybdopterin-containing nitrate reductase enzymes, has been documented in human skeletal muscle (Hellsten et al., 1996[40]; Wylie et al., 2019[124] ). In rats, XOR appears to be the major enzyme responsible for nitrate reduction in skeletal muscle, as oxypurinol, an XOR inhibitor, completely blocks this reaction in the hind legs of adult male Wistar rats (Piknova et al., 2016[82]). However, nitrate reductase activity in skeletal muscle is relatively low (Piknova et al., 2015[84]), with about 1 % of nitrate uptake converted to nitrite (Srihirun et al., 2020[100]). At 2 % oxygen, nitrate reductase activity in rat hindlimb skeletal muscle has been reported to be 0.4, 0.5, and 0.9 nmol/h/g tissue at nitrate concentrations of 100, 300, and 500 μM, respectively (Piknova et al., 2015[84]). These values are approximately 4-, 10-, and 12-fold lower than those observed in the liver, indicating that nitrate reductase activity in skeletal muscle is comparatively limited (Piknova et al., 2015[84]).

### Liver

eNOS is constitutively expressed in human hepatocytes under normal conditions, with an activity of 0.39 ± 0.14 pmol/min/mg protein in liver homogenates (McNaughton et al., 2002[69]). Constitutive expression of iNOS in the normal human hepatocytes has been reported (Leifeld et al., 2002[57]; McNaughton et al., 2002[69]), and iNOS activity in liver homogenates is 0.44 ± 0.16 pmol/min/mg protein (McNaughton et al., 2002[69]). Data regarding nNOS expression in the liver are inconclusive. Low levels of nNOS protein have been detected in mouse liver homogenates (Schild et al., 2006[94]), and selective nNOS inhibition blocks acetaminophen toxicity in mouse hepatocytes (Banerjee et al., 2015[12]). However, nNOS is not expressed in normal human hepatocytes (McNaughton et al., 2002[69]), and nNOS knockout does not alter nitrate or nitrite levels in the liver of mice (Piknova et al., 2015[84]). Previous reviews have suggested that eNOS is the principal NOS isoform contributing to NO production in hepatocytes under normal physiological conditions (Bahadoran et al., 2020[6]; Farahani et al., 2025[25]).

The liver exhibits relatively high XOR activity (Jansson et al., 2008[46]; Piknova et al., 2015[84]; Qin et al., 2012[89]; Srihirun et al., 2020[100]). In the rat liver, XOR is the primary nitrate reductase, as inhibition by oxypurinol results in approximately 51 % decrease in nitrate reductase activity (Piknova et al., 2015[84]). In mammals, nitrate reductase activity is most abundant in the gastrointestinal tract and liver, with the rank order colon > stomach > kidney > small intestine > liver > heart > lung (Jansson et al., 2008[46]). At 2 % oxygen, nitrate reductase activity in rat liver has been reported to be 1.5, 4.8, and 10.9 nmol/h/g tissue at 100, 300, and 500 μM nitrate, respectively (Piknova et al., 2015[84]). Compared with anaerobic conditions (0 % oxygen), nitrate reduction in liver homogenates decreases by approximately 25 % in the presence of 6 % oxygen, which approximates normal tissue oxygenation (~60 μM, 45 mm Hg) (Jansson et al., 2008[46]).

The characteristics of NO production and nitrate metabolism in the salivary glands, skeletal muscle, and liver are summarized in Table 1(Tab. 1) (References in Table 1: Carreau et al., 2011[18]; Frandsen et al., 1996[28]; Ghasemi, 2022[29]; Jansson et al., 2008[46]; Kusano et al., 2023[54]; Mitsui and Furuyama, 2000[71]; Piknova et al., 2015[84]; Piknova et al., 2023[85]; Piknova et al., 2024[86]; Piknova et al., 2025[83]; Qin et al., 2012[89]; Snyder et al., 1975[96]; Srihirun et al., 2020[100]; Stamler and Meissner, 2001[101]; Sugiya et al., 2001[105]; Xu et al., 1997[125]). NO production in the salivary glands is primarily NOS-dependent, with nNOS serving as the main NO-producing enzyme, and nitrate reductase activity in these glands is minimal. Skeletal muscle can generate NO via both NOS-dependent (predominantly nNOS-mediated) and NOS-independent pathways, although its nitrate reductase activity is relatively low. In the liver, eNOS is likely the principal NO-producing NOS isoform, and relatively high XOR activity enables effective conversion of nitrate to NO. mRNA expression of the XOR gene in the liver and salivary glands is approximately 330-fold and 50-fold higher, respectively, than in skeletal muscle. Nevertheless, the overall order of nitrate reductase activity is liver > skeletal muscle > salivary glands, as XOR in the salivary glands is predominantly expressed as XO (Kusano et al., 2023[54]), which is less efficient in reducing nitrite to NO than XDH (Godber et al., 2000[36]).

## Skeletal Muscle as a Major Site of Nitrate Production and Storage

Nitrate in skeletal muscle has both internal sources (oxidation of nNOS-derived NO produced via the productive cycle and NOS-derived nitrate production via the futile cycle) and external sources (uptake of dietary nitrate from the circulation) (Gilliard et al., 2018[34]; Piknova et al., 2021[87]). Oxidation of nNOS-derived NO by oxymyoglobin is the primary source of nitrate in the skeletal muscle (Piknova et al., 2016[82]). This concept is supported by evidence showing that NOS inhibition decreased nitrate levels in the hindlimb skeletal muscle of adult male Wistar rats by 67-78 % (Park et al., 2019[78]; Piknova et al., 2016[82]). In addition, nitrate levels in the hindlimb skeletal muscle of nNOS⁻/⁻ mice are lower than those of wild-type mice (Piknova et al., 2015[84]; Upanan et al., 2024[111]) by approximately 88 % (113 ± 5.8 vs. 13.2 ± 5 nmol/g (Piknova et al., 2015[84])). Oxymyoglobin, which is present at high concentrations in skeletal muscle, oxidizes NO to nitrate, whereas metmyoglobin reductase, which is also present in skeletal muscle, reduces metmyoglobin back to myoglobin (Piknova et al., 2015[84]). Nitrate levels in the skeletal muscle of myoglobin-deficient mice were 26 % lower than those of wild-type mice (Park et al., 2019[78]).

In addition to NO production via the productive cycle, nNOS can produce nitrate via the futile cycle (Stuehr et al., 2004[103]). During NO synthesis by NOS, reduction of the ferric enzyme is rate-limiting for NO production, and all NO synthesized initially binds to the ferric NOS heme (Fe^III^NO), which can proceed via two pathways: (1) dissociation of NO from Fe^III^NO (productive cycle), and (2) reduction of Fe^III^NO to ferrous heme-NO (Fe^II^NO), which releases NO very slowly and reacts with O₂ to produce nitrate (futile cycle), thereby regenerating ferric heme (Stuehr et al., 2004[103]). This mechanism indicates that NOS enzymes must balance NO dissociation and heme reduction to release synthesized NO. nNOS exists predominantly as a ferrous-NO species (Fe^II^NO), eNOS predominantly as a ferric species (Fe^III^), and iNOS occupies an intermediate position between these two extremes (Stuehr et al., 2004[103]). Therefore, NOS enzymes, in particular nNOS, can produce nitrate in tissues like skeletal muscle (Piknova et al., 2015[84]), thereby providing an intrinsic source of nitrate within the skeletal muscle (Piknova et al., 2025[83]).

Estimates indicated that baseline nitrate storage in human skeletal muscle (700 mg) exceeds that in blood (400 mg) and liver (200 mg) (Piknova et al., 2024[86]). Skeletal muscle is the largest organ in the mammalian body (Piknova et al., 2025[83]; Piknova et al., 2015[84]) with a mass of 28 kg (range: 22-36 kg) in a 70-kg man and 17 kg (range: 9.7-20.8 kg) in a 58-kg woman (Snyder et al., 1975[96]). Four reports of basal nitrate concentrations in the vastus lateralis muscle of healthy humans report values of 35 (Kadach et al., 2023[50]), 55 (Kadach et al., 2022[51]), 82 (Nyakayiru et al., 2017[73]), and 226 (Wylie et al., 2019[124]) nmol/g, yielding an average of approximately 100 nmol/g. Based on these data, the nitrate content in the skeletal muscle of young adults is estimated to be approximately 200 mg in men and 100 mg in women. However, nitrate content varies among different skeletal muscles. Using data obtained from pigs and organ/tissue weight estimates in humans, Piknova et al. proposed that human skeletal muscle stores 366-523 mg of nitrate at baseline (Piknova et al., 2024[86]). Thus, skeletal muscle accounts for approximately 20 % of total body nitrate content, representing one of the largest endogenous nitrate pools (Piknova et al., 2015[84]) and a major site of nitrate storage (Piknova et al., 2025[83]) in the human body. Its large volume, relatively low metabolic rate, relatively low nitrate reductase activity, and well-regulated blood flow make skeletal muscle an ideal site for nitrate storage for future use (Piknova et al., 2024[86]; Piknova et al., 2021[87]). Storage of nitrate in skeletal muscle may therefore act as a safeguard during temporary disruptions in dietary supply (Piknova et al., 2021[87]).

## Basal Nitrate Concentration in the Circulation, Saliva, Skeletal Muscle, and Liver

### Circulation and saliva

Substantial inter-individual differences in circulating nitrate concentrations have been reported (Bescos et al., 2025[14]; Ghasemi et al., 2008[31]; Ghasemi et al., 2010[32]; Tsikas et al., 2006[109]). Measurements of plasma nitrate in humans have yielded values ranging from 29 to 66 nmol/g (Jonvik et al., 2016[49]; Kadach et al., 2023[50]; Kadach et al., 2022[51]; Nyakayiru et al., 2017[73]; Wylie et al., 2019[124] ). Plasma nitrate levels in rats appear lower, ranging from 12.5 to 29.2 nmol/g (Ferguson et al., 2015[27]; Park et al., 2023[79]). The weighted mean plasma nitrate concentration in healthy adults is approximately 35 μM (Bahadoran et al., 2019[8]), consistent with other reports (Bahadoran et al., 2019[8]; Bescos et al., 2025[14]; Kapil et al., 2020[52]; Tsikas, 2008[108]). A systematic review and meta-analysis including data from 40 studies reported a mean fasting plasma nitrate concentration of 33.9 μM (95 % confidence interval: 29.9-37.9 μM) in healthy adult men and women (Bescos et al., 2025[14]). Overall, the average circulating nitrate concentration in healthy adults is about 35 μM, with reported values ranging from 12 to 76 μM (Ghasemi et al., 2010[32]).

Substantial inter-individual differences in basal salivary nitrate concentrations have also been reported (Bescos et al., 2025[14]; Granli et al., 1989[39]). A systematic review and meta-analysis that included data from 12 studies reported a mean fasting salivary nitrate concentration of 535.9 μM (95 % confidence interval: 384.2-687.6 μM) (Bescos et al., 2025[14]). However, basal salivary nitrate concentrations of 200 μM (Lundberg and Govoni, 2004[68]), 249 μM (Bahadoran et al., 2021[5]), and 720 μM (Govoni et al., 2008[38]) have also been reported. Overall, basal salivary nitrate concentrations in humans are approximately 500 μM, with reported values ranging from 200 to 700 μM.

At baseline, in healthy individuals, the saliva-to-plasma nitrate concentration ration has been reported to be 9 (Cortas and Wakid, 1991[20]), 10 (Bahadoran et al., 2021[5]), 16 (Bescos et al., 2025[14]), 60 (Govoni et al., 2008[38]), and 100 (Srihirun et al., 2020[100]). These findings indicate that the saliva-to-plasma nitrate concentration ratio generally ranges from approximately 10- to 100-fold (Bahadoran et al., 2021[5]; Bescos et al., 2025[14]; Cortas and Wakid, 1991[20]; Govoni et al., 2008[38]; Lundberg and Govoni, 2004[68]; Srihirun et al., 2020[100]; Weitzberg et al., 2010[119]), with values of 10-20 appearing to be reasonable estimates (Weitzberg et al., 2010[119]).

### Skeletal muscle

Table 2(Tab. 2) (References in Table 2: Gilliard et al., 2018[34]; Ibrahim and Ashour, 2011[44]; Kadach et al., 2022[51]; Kadach et al., 2023[50]; Long et al., 2020[63]; Nyakayiru et al., 2017[73]; Park et al., 2019[78]; Park et al., 2021[80]; Park et al., 2023[79]; Piknova et al., 2015[84]; Piknova et al., 2016[82]; Piknova et al., 2023[85]; Piknova et al., 2024[86]; Troutman et al., 2018[107]; Upanan et al., 2024[111]; Wylie et al., 2019[124] ) summarizes basal nitrate content in different skeletal muscles as measured in humans (Kadach et al., 2023[50]; Kadach et al., 2022[51]; Nyakayiru et al., 2017[73]; Wylie et al., 2019[124]), rats (Gilliard et al., 2018[34]; Ibrahim and Ashour, 2011[44]; Long et al., 2020[63]; Park et al., 2023[79]; Park et al., 2021[80]; Piknova et al., 2016[82]; Piknova et al., 2015[84]; Piknova et al., 2023[85]; Troutman et al., 2018[107]), mice (Park et al., 2019[78]; Piknova et al., 2015[84]; Upanan et al., 2024[111]), and pigs (Piknova et al., 2024[86]); values presented graphically were extracted as described previously (Gheibi et al., 2019[33]). In humans, all measurements have been conducted in the vastus lateralis, yielding basal nitrate concentrations ranging from 35 to 226 nmol/g. Most studies have been conducted in rats, reporting values ranging from 12 to 222 nmol/g, with the lowest concentrations observed in the tibialis anterior (TA) and the highest in the soleus. Studies in mice using mixtures of hindlimb muscles reported concentrations ranging from 17 to 113 nmol/g, whereas the only study in pigs reported a concentration of 103 nmol/g. Considerable variation exists in basal nitrate levels both within the same muscle among different individuals and among different skeletal muscles. In healthy young adults, large inter-individual variability in baseline muscle nitrate concentration has been reported, with values ranging from 30 to 1,036 nmol/g tissue (Wylie et al., 2019[124]). In addition, nitrate content in the gluteus muscle (43.4 nmol/g) was 3.6-fold higher than in TA (12.1 nmol/g) (Park et al., 2021[80]), indicating that different skeletal muscles store different amounts of nitrate (Park et al., 2021[80]; Piknova et al., 2021[87]). Despite this variability, an approximate average value of 100 nmol/g (range: 12-226 nmol/g) can be considered for overall skeletal muscle nitrate concentration.

Differences in nitrate concentration among skeletal muscles may be related to differences in predominant muscle fiber type (Kadach et al., 2022[51]; Park et al., 2021[80]), differential expression of nitrate transporters, differences in nitrate production capacity, and variation in XOR expression or activity (Piknova et al., 2021[87]). In rats, the percentages of type II fibers in the extensor digitorum longus (EDL), gluteus, TA, vastus lateralis, gastrocnemius, and soleus are 100 %, 100 %, 99.6 %, 98.8 %, 94 %, and 20 %, respectively (Eng et al., 2008[23]). Species differences are also evident: in rats, the vastus lateralis muscle contains approximately 98.8 % type II fibers and only 1.2 % type I fibers (Eng et al., 2008[23]), whereas in humans, the vastus lateralis contains approximately 40 % and 44 % type I fibers in healthy young men and women, respectively (Staron et al., 2000[102]). In rats, higher nitrate concentrations have been observed in skeletal muscles with a higher proportion of type I (slow-twitch) fibers compared with type II (fast-twitch) fibers, with nitrate content in the soleus muscle being 3.5-fold higher than in the vastus lateralis (Long et al., 2020[63]). In addition, basal microvascular PO_2_ was higher in soleus (32 ± 3 mm Hg) than in gastrocnemius (24 ± 2 mm Hg) in rats (Ferguson et al., 2015[27]). However, this relationship requires further investigation, as the opposite pattern has also been reported, with nitrate content in the gluteus muscle being 1.9-fold higher than in the soleus muscle (Park et al., 2021[80]).

As shown in Table 2(Tab. 2), the skeletal muscle-to-plasma ratio of nitrate varies across skeletal muscles, with reported values ranging from below 1 to above 1. In humans, four studies conducted in the vastus lateralis muscle yield a mean skeletal muscle-to-plasma nitrate ratio of approximately 2.1. In rats, reported ratios are 1.3 for the TA and gastrocnemius muscles, 1.4 for the EDL, 2.2 for the vastus lateralis, 2.3 for the gluteus, and 3.8 for the soleus muscle.

Overall, nitrate content in skeletal muscle varies substantially across muscle types and species, an issue that warrants further investigation.

As shown in Table 3(Tab. 3) (References in Table 3: Gilliard et al., 2018[34]; Park et al., 2019[78]; Park et al., 2021[80]; Park et al., 2023[79]; Piknova et al., 2015[84]; Piknova et al., 2016[82]; Piknova et al., 2023[85]; Piknova et al., 2024[86]; Upanan et al., 2024[111]), nitrate content in the liver has been measured in rats (Gilliard et al., 2018[34]; Park et al., 2023[79]; Park et al., 2021[80]; Piknova et al., 2016[82]; Piknova et al., 2015[84]; Piknova et al., 2023[85]), mice (Park et al., 2019[78]; Piknova et al., 2015[84]; Upanan et al., 2024[111]), and pigs (Piknova et al., 2024[86]). Reported values in rats range from 4.7 to 12.7 nmol/g, which are similar to those measured in mice (7.4-16.9 nmol/g) and pigs (15.7 nmol/g) (Piknova et al., 2024[86]). One study reported higher nitrate levels in rat liver (46.8 ± 9.0 nmol/g tissue) (Gilliard et al., 2018[34]). In addition, the liver-to-plasma nitrate ratio is approximately 0.24 in rats, 0.40 in pigs, and 0.48 in mice. Overall, a reasonable estimate of hepatic nitrate content is approximately 10 nmol/g (range: 5-15 nmol/g), with the liver-to-plasma nitrate ratio being consistently below 1 across all species studied.

## Nitrate Gradient from Skeletal Muscle to Circulation to Liver

A nitrate gradient from skeletal muscle (the site of nitrate synthesis and storage) to blood and, subsequently, to the liver (the site of nitrate reduction) has been proposed (Kadach et al., 2023[50]; Nyakayiru et al., 2020[74]; Piknova et al., 2015[84]; Piknova et al., 2021[87]). This hypothesis is supported by experimental evidence obtained in pigs (Piknova et al., 2024[86]), rats (Park et al., 2023[79]; Park et al., 2021[80]; Piknova et al., 2015[84]), and humans (Wylie et al., 2019[124]). In pigs, basal nitrate concentrations in the gluteus skeletal muscle (103.5 ± 24.6 nmol/g tissue) were higher than those in plasma (39.8 ± 19.7 nmol/g tissue) and liver (15.7 ± 5.2 nmol/g tissue) (Piknova et al., 2024[86]), yielding a skeletal muscle-to-plasma-to-liver ratios of 2.6:1:0.4. In rats, basal nitrate concentration in the gluteus skeletal muscle (28.2 nmol/g) was higher than in plasma (12.5 nmol/g) and liver (8.1 nmol/g) (Park et al., 2023[79]), yielding a ratio of 2.3:1:0.29. Another study of the gluteus skeletal muscle in rats reported similar ratios of 2.3:1:0.25 (Park et al., 2021[80]).

Nitrate concentration in rat hindlimb skeletal muscle (212.4 ± 52.1 nmol/g tissue) was approximately threefold higher than in blood (76.6 ± 2.6 nmol/g tissue) and approximately seventeenfold higher than in liver (12.7 ± 4.6 nmol/g tissue) (Piknova et al., 2015[84]), resulting in a ratio of 2.8:1:0.17. In humans, basal nitrate concentration in skeletal muscle is approximately 2-4-fold higher than in plasma, as documented in the vastus lateralis muscle of healthy young adults (81.9 vs. 38.1 μM (Nyakayiru et al., 2017[73]) and 226 ± 213 vs. 54 ± 27 nmol/g tissue (Wylie et al., 2019[124])).

**Figure 5:**
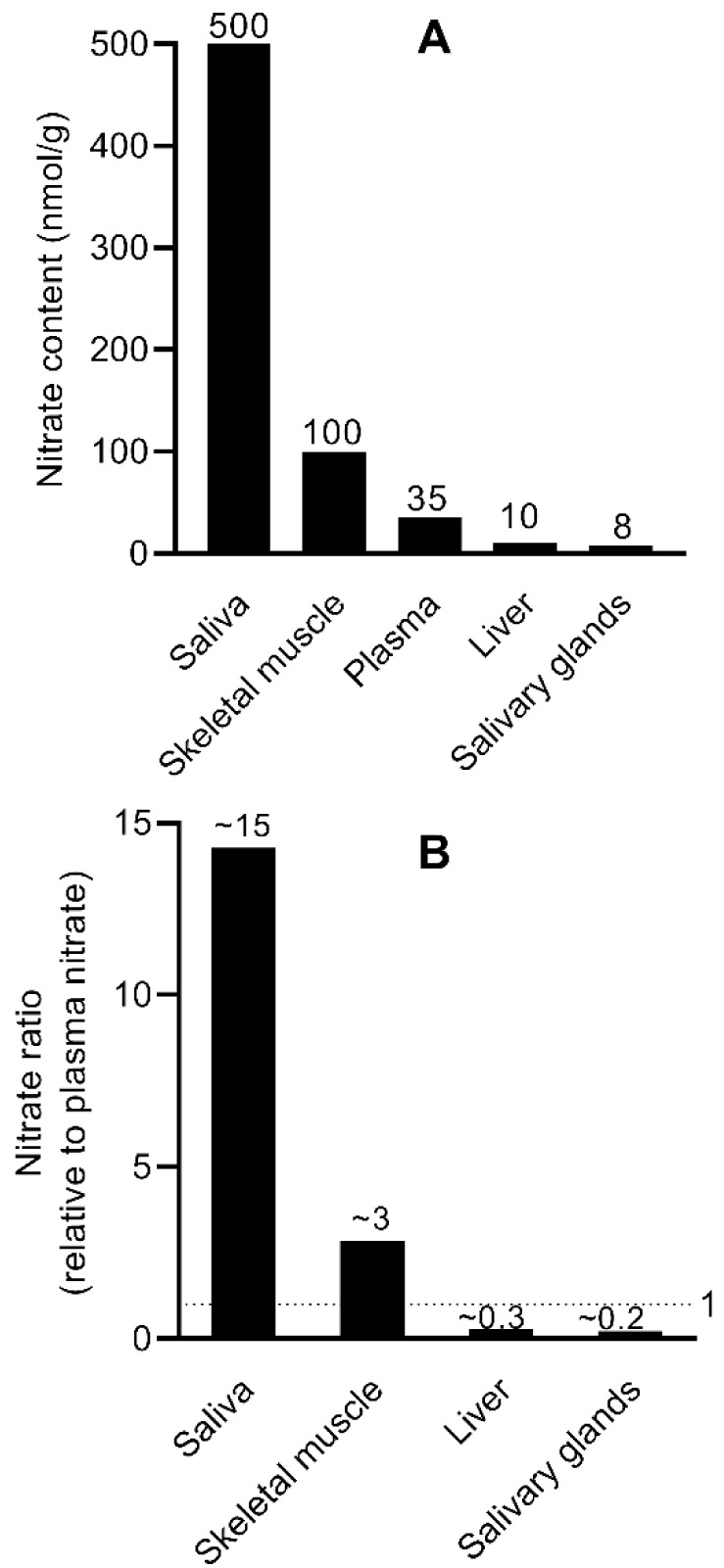
Basal nitrate content in saliva, skeletal muscle, plasma, liver, and salivary glands (A); Ratios of nitrate content in saliva, skeletal muscle, liver, and salivary glands relative to plasma nitrate (B)

As shown in Figure 5(Fig. 5), basal nitrate concentrations in the saliva (500 nmol/g) and skeletal muscle (100 nmol/g) are approximately 15-fold and 3-fold higher than plasma (35 nmol/g). In contrast, nitrate contents in the liver (10 nmol/g) and salivary glands (8 nmol/g) are only 0.3- and 0.2-fold that of plasma, respectively. Alternatively, when plasma nitrate concentration is used as the reference, nitrate ratios of 1:15:3:0.3:0.2 are obtained for plasma, saliva, skeletal muscle, liver, and salivary glands, respectively. Collectively, these data support the presence of a nitrate gradient from skeletal muscle to plasma to liver (approximately 3:1:0.3).

## Nitrate Concentration in the Saliva, Skeletal Muscle, and Liver after Nitrate Ingestion

Ingestion of nitrate increases nitrate content in the plasma (Bahadoran et al., 2021[5]; du Toit et al., 2024[22]; Jonvik et al., 2016[49]; Kadach et al., 2023[50]; Kadach et al., 2022[51]; Lundberg and Govoni, 2004[68]; Pannala et al., 2003[77]; Webb et al., 2008[117]; Wylie et al., 2013[123]; Wylie et al., 2019[124]), saliva (Bahadoran et al., 2021[5]; du Toit et al., 2024[22]; Govoni et al., 2008[38]; Kadach et al., 2023[50]; Kadach et al., 2022[51]; Lundberg and Govoni, 2004[68]; Pannala et al., 2003[77]; Webb et al., 2008[117]), skeletal muscle (Gilliard et al., 2018[34]; Kadach et al., 2022[51]; Nyakayiru et al., 2017[73]; Park et al., 2023[79]; Piknova et al., 2023[85]; Piknova et al., 2024[86]; Wylie et al., 2019[124]), and liver (Gilliard et al., 2018[34]; Park et al., 2023[79]; Piknova et al., 2023[85]; Piknova et al., 2024[86]).

### Circulation and saliva

Table 4(Tab. 4) (References in Table 4: Bahadoran et al., 2021[5]; du Toit et al., 2024[22]; Govoni et al., 2008[38]; Jonvik et al., 2016[49]; Kadach et al., 2022[51]; Kadach et al., 2023[50]; Lundberg and Govoni, 2004[68]; Pannala et al., 2003[77]; Webb et al., 2008[117]; Wylie et al., 2013[123]; Wylie et al., 2019[124]) summarizes increases in plasma and salivary nitrate concentrations following nitrate ingestion in healthy humans. Interventions includes sodium nitrate (Govoni et al., 2008[38]; Jonvik et al., 2016[49]; Lundberg and Govoni, 2004[68]), potassium nitrate (du Toit et al., 2024[22]; Kadach et al., 2023[50]; Kadach et al., 2022[51]), or nitrate-rich foods such as beetroot (Bahadoran et al., 2021[5]; Webb et al., 2008[117]; Wylie et al., 2013[123]; Wylie et al., 2019[124]), lettuce (Pannala et al., 2003[77]), and green leafy vegetables (du Toit et al., 2024[22]). Nitrate doses ranged from 3.1 to 21.5 mg/kg, and total nitrate intake ranged from 222 to 1,395 mg/day.

Human studies indicate that following nitrate ingestion, plasma nitrate concentration increases by approximately 2-20-fold and typically peaks at around 90 min (range: 30-160 min). In addition, salivary nitrate concentration increases by approximately 3- to 85-fold and generally peaks at around 90 min (range: 30-180 min). Data presented in Table 4(Tab. 4) indicate that following nitrate ingestion, plasma and salivary nitrate concentrations peak simultaneously (at approximately 90 min), consistent with a previous report (Lundberg and Govoni, 2004[68]). However, findings on this issue are not entirely consistent. Some studies have reported that plasma nitrate peaks before salivary nitrate (Kadach et al., 2023[50]; Pannala et al., 2003[77]; Webb et al., 2008[117]), whereas others have reported that plasma nitrate peaks after salivary nitrate (Bahadoran et al., 2021[5]; Kadach et al., 2022[51]).

Increased plasma nitrate concentrations following nitrate consumption have also been reported in animal studies, including beetroot supplementation (62 mg/kg for 5 days) (Ferguson et al., 2015[27]) and sodium nitrate administration (1g/L in drinking water for 5 days) (Piknova et al., 2023[85]) in rats, resulting in 2.7-fold (29 ± 6 to 79 ± 17 nmol/g) and 3.9-fold (15.1 ± 3.9 to 58.8 ± 30.8 nmol/g) increases, respectively. In pigs, ingestion of Na¹⁵NO₃ (9.3 mg/kg, single dose) increased plasma nitrate concentration approximately 5-fold (39.8 ± 19.7 to 199 ± 35.6 μM) (Piknova et al., 2024[86]).

**Figure 6:**
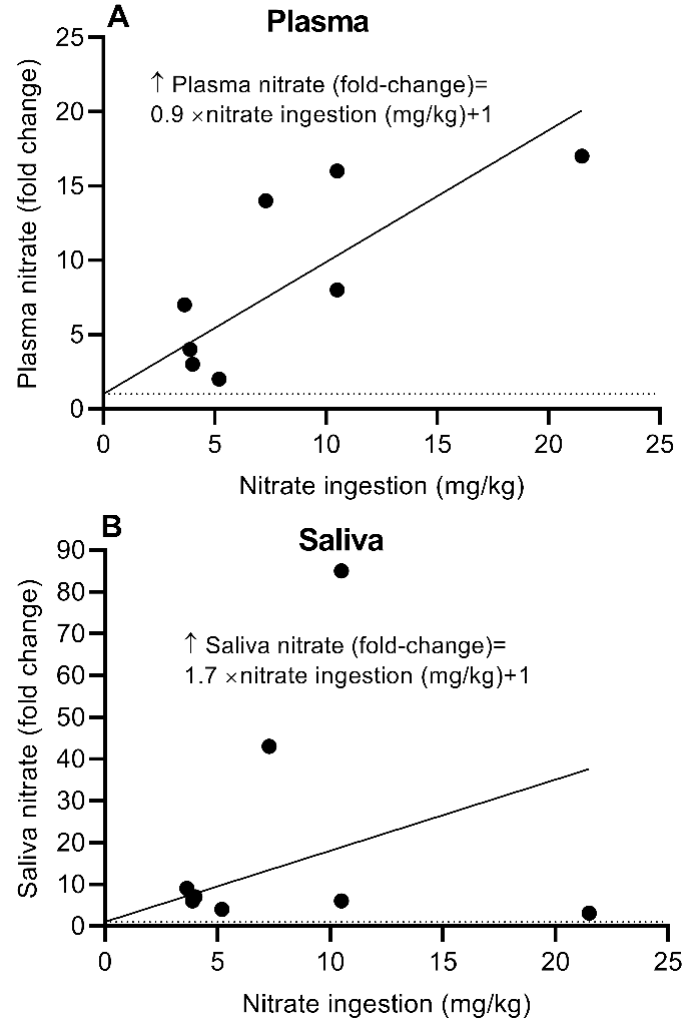
Changes in plasma (A) and saliva (B) nitrate concentrations following ingestion of different amounts of nitrate in humans. Horizontal dot lines indicate basal nitrate concentrations in plasma (33 nmol/g) and saliva (724 nmol/g).

It has been shown that plasma nitrate concentration increases from a baseline value of 34 ± 8 μM by 4-, 8-, and 17-fold following ingestion of 3.1, 6.8, and 13.6 mg/kg nitrate from beetroot, respectively, at 2.5 h post-ingestion in healthy men (Wylie et al., 2013[123]). Figure 6(Fig. 6) illustrates changes in plasma and salivary nitrate concentrations as a function of ingested nitrate dose in humans, based on studies in which both circulating and salivary nitrate concentrations were measured simultaneously (Bahadoran et al., 2021[5]; du Toit et al., 2024[22]; Kadach et al., 2023[50]; Kadach et al., 2022[51]; Lundberg and Govoni, 2004[68]; Pannala et al., 2003[77]; Webb et al., 2008[117]). Increased plasma and salivary nitrate concentrations following beetroot ingestion are dose-dependent; for example, when an individual consumes 10 mg/kg nitrate, circulating nitrate concentration increases by approximately 10-fold, whereas salivary nitrate concentration increases by approximately 18-fold.

### Skeletal muscle

Nitrate ingestion increases skeletal muscle nitrate content in humans (Kadach et al., 2022[51]; Wylie et al., 2019[124] ) and animals (Gilliard et al., 2018[34]; Piknova et al., 2023[85]; Piknova et al., 2024[86]) by approximately 3-5-fold and 1-2-fold, respectively (Table 5(Tab. 5); References in Table 5: Gilliard et al., 2018[34]; Kadach et al., 2022[51]; Kadach et al., 2023[50]; Park et al., 2023[79]; Piknova et al., 2023[85]; Piknova et al., 2024[86]; Wylie et al., 2019[124]). Studies employing a single dose of nitrate ingestion measured skeletal muscle nitrate concentrations at 2-3 h post-ingestion (Kadach et al., 2022[51]; Piknova et al., 2024[86]; Wylie et al., 2019[124]). A study by the Piknova group indicates that increases in skeletal muscle nitrate content following nitrate ingestion vary among muscle types, with the TA and EDL showing the lowest and highest increases, respectively (Piknova et al., 2023[85]).

### Liver

Animal studies indicate that liver nitrate content increases following nitrate ingestion in rats (Gilliard et al., 2018[34]; Park et al., 2023[79]; Piknova et al., 2023[85]) and pigs (Piknova et al., 2024[86]) by about 2-3-fold (Table 6(Tab. 6); References in Table 6: Gilliard et al., 2018[34]; Park et al., 2023[79]; Piknova et al., 2023[85]; Piknova et al., 2024[86]).

## Conclusion and Future Perspective

According to the compartmentalization model of nitrate homeostasis, excess NO produced by NOS enzymes is converted to nitrate and stored in specific tissues, thereby ensuring rapid and uninterrupted access to NO. Human and animal studies indicate the existence of a nitrate gradient from skeletal muscle to plasma to liver (3:1:0.3). Of the approximately 3 g of nitrate present in the human body, about 20 % is stored in skeletal muscle, which represents one of the largest pools of endogenous nitrate and a major site of nitrate storage in humans. A higher skeletal muscle-to-plasma nitrate ratio, together with the large nitrate pool in skeletal muscle, favors the release of nitrate from skeletal muscle into the circulation when needed. In contrast, the liver-to-plasma nitrate ratio is < 1, indicating net uptake of nitrate by the liver from plasma. Indeed, experiments in pigs have demonstrated that the liver takes up nitrate from the circulation at approximately 2 μmol/min (Eriksson et al., 2018[24]). Nitrate ingestion increases nitrate content in plasma, skeletal muscle, and liver; increases in tissue nitrate following dietary nitrate supplementation are almost exclusively attributable to the introduction of exogenous nitrate into the body (Kadach et al., 2023[50]).

Despite the explanatory value of the compartmentalization model of nitrate homeostasis, several important issues remain to be investigated. First, nitrate transport across tissues is considered without a complete understanding of the mechanisms governing nitrate movement across cellular membranes. Although several proteins, including sialin (Qin et al., 2012[89]), aquaporin 6 (AQP6) (Ikeda et al., 2002[45]), Chloride channels (ClC-1) (Rychkov et al., 1998[93]; Srihirun et al., 2020[100]), and sodium-iodide symporter (NIS) (Picozzi et al., 2026[81]), have been identified as potential nitrate transporters in mammals, the exact mechanisms underlying nitrate transport remain to be determined. Second, the compartmentalization model of nitrate homeostasis should be expanded to incorporate the roles of additional tissues and organs, such as skin and bone (Piknova et al., 2024[86]) or the lung (Eriksson et al., 2018[24]) as nitrate storage sites, as well as the kidney (Eriksson et al., 2018[24]) as a potential nitrate reduction site. Third, skeletal muscles are heterogeneous, and animal studies indicate that different muscles vary in their capacity for nitrate storage and release (Piknova et al., 2023[85]). Accordingly, further studies are required to clarify the contribution of individual muscles to overall nitrate homeostasis. In particular, all measurements of nitrate content in human skeletal muscle to date have been conducted in the vastus lateralis, highlighting the need for data from other muscle groups.

In conclusion, the compartmentalization model of nitrate homeostasis provides a partial framework for understanding whole-body NO homeostasis and may be particularly relevant to physiological and pathological conditions in which NO plays an important role, including exercise physiology, metabolic disorders, and liver pathophysiology.

## Notes

Sajad Jeddi and Khosrow Kashfi (Department of Molecular, Cellular, and Biomedical Sciences, Sophie Davis School of Biomedical Education, City University of New York School of Medicine, New York, NY 10031, USA; E-mail: drkho@verizon.net) contributed equally as corresponding author.

## Declaration

### Acknowledgments

This study was supported by Shahid Beheshti University of Medical Sciences (Grant number: 43018291-1), Tehran, Iran. KK is supported in part by the National Institutes of Health, USA, grant number 2U54MD017979-01A1.

### Conflict of interest

The authors declare that they have no conflict of interest.

### Artificial Intelligence (AI) - assisted technology

Artificial intelligence was not used in the preparation of this manuscript.

## Figures and Tables

**Table 1:**
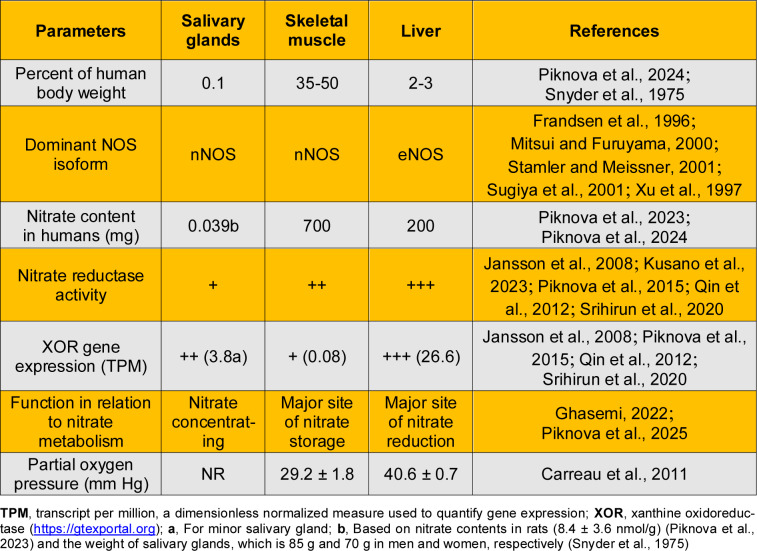
Characteristics of salivary glands, skeletal muscles, and the liver in relation to nitric oxide (NO) production and nitrate metabolism

**Table 2:**
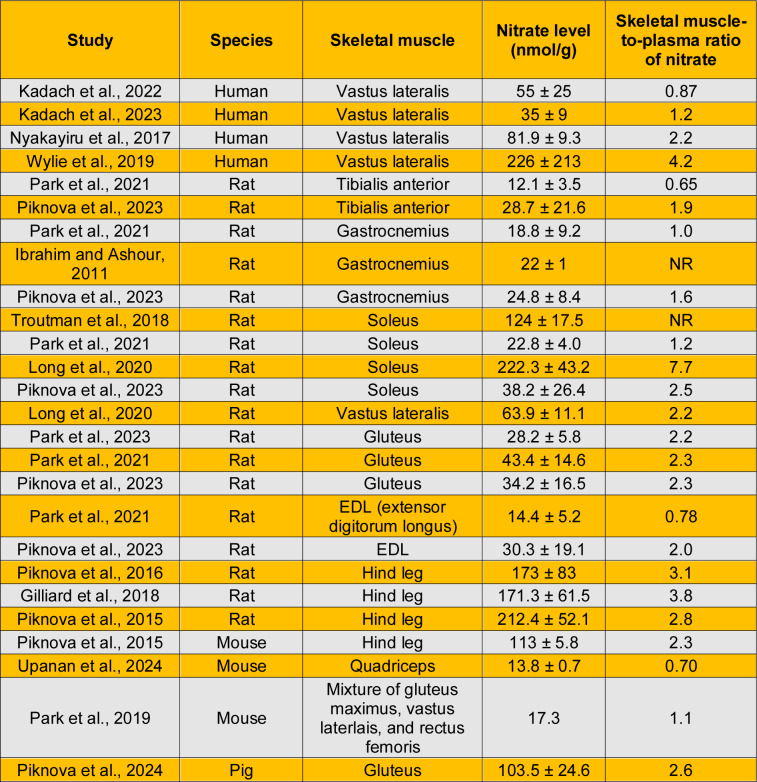
Basal nitrate content and skeletal muscle-to-plasma ratio of nitrate in different skeletal muscles

**Table 3:**
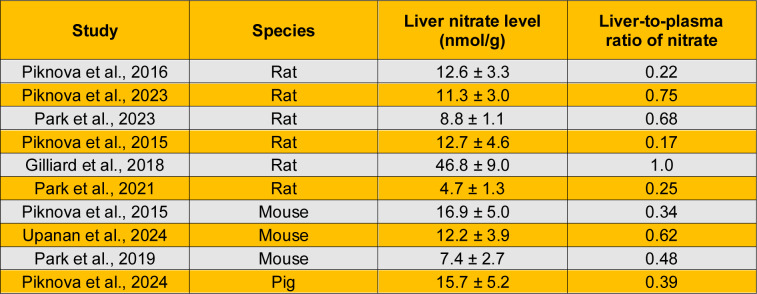
Basal nitrate content in the liver and liver-to-plasma ratio of nitrate

**Table 4:**
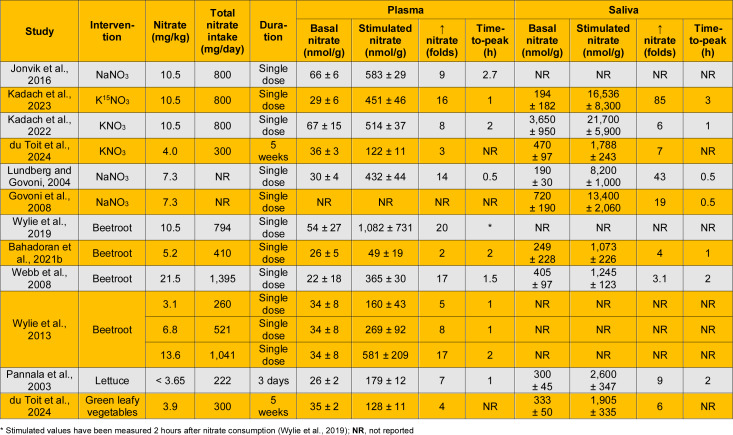
Increased plasma and salivary nitrate concentrations following nitrate ingestion in healthy humans

**Table 5:**
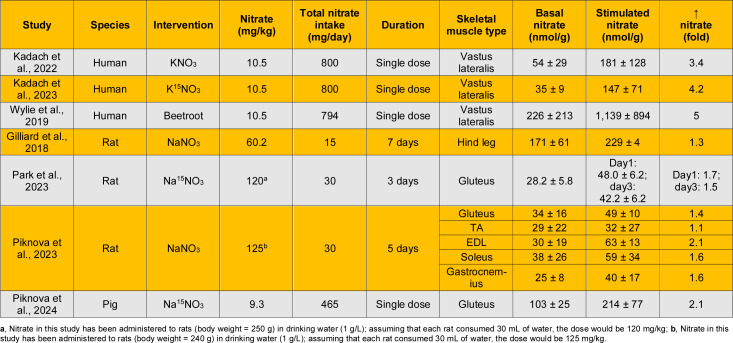
Increased skeletal muscle nitrate concentrations following nitrate ingestion in healthy humans and animals

**Table 6:**
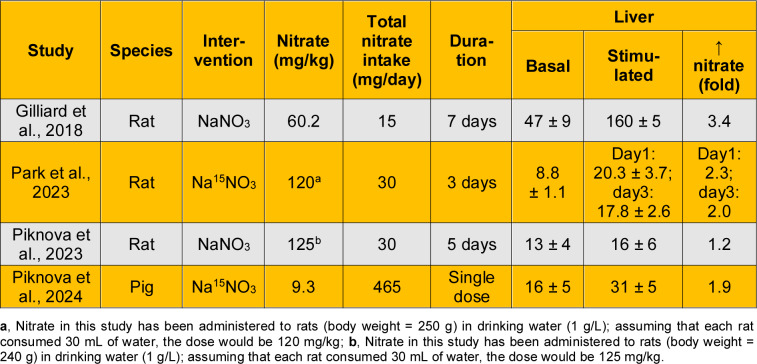
Increased liver nitrate concentrations following nitrate ingestion in healthy animals

**Figure 1:**
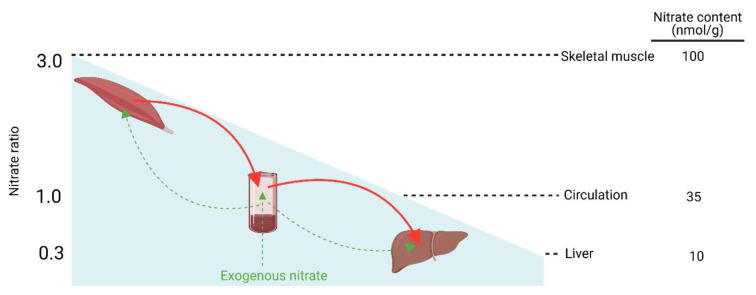
Graphical abstract: Compartmentalization model of nitrate homeostasis. Nitrate concentrations in skeletal muscle, plasma, and liver are about 100, 35, and 10 nmol/g, respectively, corresponding to a skeletal muscle-to-plasma-to-liver ratio of ~3:1:0.3. With exogenous nitrate consumption, skeletal muscle stores nitrate. When there is no exogenous supply, nitrate can move from skeletal muscle to plasma and then to liver.
